# A Systematic Review of Financial Debt in Adolescents and Young Adults: Prevalence, Correlates and Associations with Crime

**DOI:** 10.1371/journal.pone.0104909

**Published:** 2014-08-19

**Authors:** Machteld Hoeve, Geert Jan J. M. Stams, Marion van der Zouwen, Margaretha Vergeer, Kitty Jurrius, Jessica J. Asscher

**Affiliations:** 1 Research Institute of Child Development and Education, University of Amsterdam, Amsterdam, The Netherlands; 2 Kohnstamm Institute, Amsterdam, The Netherlands; 3 Alexander Foundation, Amsterdam, The Netherlands; Center of nonlinear, China

## Abstract

Financial debt in young people has increased in recent years. Because debt may have severe consequences, and it may enhance criminal behavior, insight into the prevalence and determinants of debt and its association with crime is important. We conducted a systematic review and meta-analysis of 36 manuscripts to examine the prevalence of financial debt (*k* = 23), correlates and risk factors of debt (*k* = 16), and associations between debt and criminal behavior in adolescents and young adults (*k* = 8). Findings revealed that the prevalence of debt is substantial among young people; on average, 49% reported to have at least some debt, 22% had financial problems. Older participants and ethnic minorities were found to have higher levels of debt than younger and indigenous counterparts. Females had more financial problems and higher student loans. Low self-esteem, a pro-debt attitude (of young people and their parents), lack of perceived control towards financial management, poor social functioning, financial stress and external locus of control were found to have the strongest associations with debt. Studies reported strong associations between debt and crime. Particularly, strong associations were found between serious and persistent crime in young people and later (young adult) debt or financial problems.

## Introduction

Previous research showed that debt and financial problems of adolescents and young adults in Western countries have increased over time [1]–[3]. For example, in the U.S.A., young adults with a credit card spent on average almost a fourth of their income servicing debt in 2004 compared to about a fifth in 1992 [4]. Young adult students are at risk to have debt due to a rise in college costs. In the last decade, student loans have increased together with tuition fees [5]. The use of mobile phones has put adolescents at increased risk for debt [6]. In Europe, a similar trend has been observed in that in the past two decades consumer borrowing and saving behavior have significantly been changed; Europeans tend to borrow more often and save less than they used to do [1].

Research has revealed several harmful consequences of financial debt. For example, in a sample of young adults some evidence was found to suggest that credit card debt has negative consequences on a sense of mastery and the level of self-esteem over time, possibly because financial stress adds up as young people age [2]. Other studies showed that debt is associated with lower levels of happiness and well-being [7] and with poorer mental and physical health conditions [8] in students. Further, financial debt in young adults has found to be associated with lower levels of academic success, poorer life satisfaction, depressed mood and poorer physical health [9]. Thus, not only can debt be problematic in itself, but it is also found to be related to other problematic outcomes for the individual.

Another problem among young people, which is very vexing, is criminal behavior. The incidence of criminal behavior is relatively high among (late) adolescents and young adults [10]. Several scholars have suggested that financial debt and criminal behavior are related. For example, Merton [11] and Agnew [12] explained criminal behavior from the perspective of *strain*. In short, according to Merton [11] crime is a consequence of discrepancies between needs and desires on the one hand and opportunities and expectations to reach these needs in a legitimate way on the other hand. This theory has mainly bearing on individuals with a low socio-economic status (SES), who generally have less resources and opportunities to reach their goals. The assumption is that strain between desires and chances to fulfill these desires might lead to criminal behavior. Applying these theoretical notions to debt and criminal behaviors, we assume that if young people have debt or financial problems they have less access to material goals, and this could result in delinquency in order to fulfill their desires. Thus, it can be hypothesized that debt and financial problems in young people increase the risk for criminal behavior.

Alternatively, in Gottfredson and Hirschi's [13] general theory of crime, which attributes engaging in crime to a lack of self-control, self-control is shaped in childhood by various factors, such as parenting. When children have developed relatively low levels of self-control by middle childhood, this latent criminal propensity remains relatively stable during the life course. A lack of self-control may not only cause criminal behavior, but also other types of analogous risk behaviors aimed at immediate gratification. Jessor [14] postulated that various risk behaviors among youth - such as delinquency, drug use, school dropout and general deviant behavior - can be considered as a risky life style. Involvement in any one of these behaviors will likely increase the risk for involvement in other problem behaviors, because these risk behaviors share a similar etiology. It is therefore possible that both debt and delinquent behavior are risk taking behaviors that have a similar origin. On the basis of these models financial debt and crime are associated.

Given the rise in debt and financial problems among youth, the worsened economic conditions of Western countries, and detrimental consequences of debt, it is important to gain insight into a) the rate of financial debt in young people, b) which factors put young people at risk for financial debt, and c) the extent to which empirical evidence exists for the association between financial debt and crime in adolescents and young adults. Risk factors of debt are relevant for understanding which factors explain that some adolescents or young adults are more likely to borrow money or have financial problems than others. This knowledge is in turn useful for prevention or intervention purposes. Therefore, a review of financial debt in young people is warranted. Given that the incidence of criminal behavior is relatively high among (late) adolescents and young adults, and financial resources and knowledge are relatively scarce among these age groups, it is important to gain more insight into financial debt in young people and the co-occurrence of debt and crime in this group.

Several empirical studies have investigated the prevalence of debt, risk factors of debt, associations with criminal behavior, and correlates of debt, ranging from financial knowledge [15] to parental support [16]. To our knowledge, a review of financial debt in adolescents and young adults has not been conducted. The current systematic review examined the following research questions: 1) what is the prevalence of financial debt in adolescents and young adults? 2) What are significant risk factors or correlates of debt? In other words, which young people are more likely to have debt? We will focus on a broad range of correlates that range from demographic characteristics, characteristics related to financial management and knowledge, and social factors such as parental support and peer pressure. 3) Is debt associated with criminal behavior in young people? Although some research suggest that debt and crime in young people is associated, we aim to gain more insight in the strength of the association and focus on potential differences between types of offenders and types of debt.

## Methods

We conducted a systematic review of the literature applying meta-analytic techniques in order to be able to quantitatively synthesize empirical results across studies. Effect sizes were calculated for each correlate or risk factor of debt. The same was done for the association between delinquency and debt. Also, we conducted moderator analyses in order to examine to what extent effect sizes varied by sample and study characteristics. We found a fair amount of studies reporting on the prevalence of debt, and therefore calculated a mean effect size for the proportion of debt, and conducted moderator analyses in order to examine which sample and study characteristics were associated with the proportion of debt.

The tendency of journals to accept papers that report strong significant associations, referred to as publication bias, may have serious implications for the final conclusions of systematic reviews [17], [18]. Rosenthal [19] identified this as the file drawer problem, which refers to the problem that many unpublished studies exist and that the overall results may be different from those that are published. Several methods exist to address potential effects of publication bias, but each has its own shortcoming [20]. Following the advice of Rothstein [20], we apply two of the conventional methods that address publication bias. First, we examined what effect publication bias could have on the meta-analytic results by inspecting the distribution of each individual study's effect size on the horizontal axis against its sample size, standard error or precision (the reciprocal of the standard error) on the vertical axis. The distribution of effect sizes should be shaped as a funnel if no publication bias is present, since the more numerous studies with small sample sizes are expected to show a larger variation in the magnitude of effect sizes than the less numerous studies with large effect sizes. We checked funnel plots for categories of effect sizes with at least 10 independent effect sizes. Second, we provide a fail-safe number, which estimates the number of unretrieved studies averaging null results needed to bring the overall medium or large combined effect size at a small or medium level [22]. The best solution, however, is to try to prevent effects of publication bias by obtaining unpublished material [17], [21]. Therefore, the present systematic review includes published studies, including journal articles, books and book sections as well as unpublished reports and dissertations.

### Inclusion criteria and search strategy

The following selection criteria were used. First, studies had to focus on (problematic or non-problematic) debt, loans, borrowing behavior, credit or financial problems in adolescents or young adults. Studies that focused on financial problems of parents or other family members were not included. Second, studies had to focus on debt in relation to a) risk factors or correlates of debt and b) delinquency. We considered delinquency as behavior prohibited by the law, such as behaviors ranging in seriousness from petty crime and vandalism to serious violence and murder. Studies that focused exclusively on problem behaviors that are not prohibited by the law were not included. Third, studies had to focus on adolescents (about ages 12–18) or young adults (about 18–30). Fourth, given that financial problems of youths in Western countries have increased [1] and that debt might play a different role in non-Western societies, we focus on studies from Western countries. Finally, manuscripts were included where bivariate associations with (or proportions of) debt were reported, as multivariate results cannot be compared across studies [22].

Studies were collected according to the following procedure. First, electronic databases, including Academic search premier, Business Search Premier, EconLit, ERIC, PsycINFO, and Sociological Abstracts, were searched through for articles, books, chapters, reports, theses and reviews. We used a variety of terms related to debt and crime. Search terms related to debt (debt*, indebtedness, over-indebtedness, credit, loan, borrow, or financial problems) were cross-referenced with terms related to age group (adolescen*, youth, juvenile*, young, or student), correlates (risk factor, correlate*, cause, relation* or association) or criminal behavior (delinq*, crim*, or offend*). We considered the concept of debt in a rather broad way, focusing on both problematic and non-problematic debt and financial problems. In order to find risk factors for debt, we cross-referenced the terms related to debt with terms related to age group and correlates. In our search for studies examining the relation between debt and delinquency, we cross-referenced the terms related to debt with terms related to age group and delinquent behavior. We searched in abstracts of manuscripts. In addition, we used these terms to search in Google (Scholar) in order to find more unpublished material, such as reports on empirical studies and theses. Finally, manual searches were applied, which means that reference lists of reviews and other articles were checked in order to find relevant studies not found in the electronic databases. We screened 841 abstracts and assessed full-texts of 90 manuscripts (see Figure S1). We excluded 54 manuscripts because the analyses reported were multivariate (*k* = 4) or qualitative (*k* = 2), the subjects were adults (*k* = 11) or the focus was on variables related to finances other than debt such as socio-economic status, financial problems of the family, poverty, unemployment, and dissatisfaction about income (*k* = 34). We eventually found 36 manuscripts that met our criteria (see Table 1).

**Table 1:** Description of Major Characteristics of Studies Used in the Present Review.

Reference	Publication type	Research design	Sample type, sex, age	Sample size	Country	Relevant data	Results
Archuleta, Dale & Spann [50]	Journal article (peer reviewed)	Quantitative, Cross-sectional	Students receiving services at fin. counseling center, Mixed, Mean age 23.8 years	*N* = 180	USA	Correlates of debt	Financial anxiety, gender (being female), financial satisfaction, and financial knowledge (less) is associated with debt. Gross income, marital status and ethnicity is not related to financial debt.
Blom, Weijters, & Van der Laan [29]	Factsheet	Quantitative, Cross-sectional	General population, Mixed, Ages 10–17	*N* = 2116	Netherlands	Proportion of debt, Debt-crime association	Financial problems were associated with self-reported delinquency. For older participants the financial problem-delinquency association was stronger than for younger participants. For boys the association between financial problems and vandalism was stronger than for girls.
Caputo [51]	Journal article (peer reviewed)	Quantitative, Longitudinal	General population, Mixed, Ages 20–28 (at first debt measurement)	*N* = 5304	USA	Proportion of debt, Correlates of debt	Health status and level of changes in income were associated with debt; age and ethnicity were associated with short-term and intermittent debt; locus of control, family structure during adolescence, SES, work effort, and marital status were associated with intermittent and chronic debt; self-esteem, sex, SES, and work effort were associated with chronic debt.
Crocker & Luhtanen [27]	Journal article (peer reviewed)	Quantitative, Longitudinal	Students, Mixed, Age 18	*N* = 631	USA	Correlates of debt	Lower self-esteem, neuroticism, GPA is associated with financial problems. Narcissism, agreeability, extraversion, conscientiousness, and openness is not associated with financial problems.
Davies & Lea [52]	Journal article (peer reviewed)	Quantitative, Cross-sectional	Students, Mixed, Ages 18–21	*N* = 140	UK	Proportion of debt, Correlates of debt	Students with debt were older, more often male, had a more positive attitude towards debt, had different types of debt and were less worried about the level of their bank account. Students in 2nd or 3rd year of university (compared to 1^st^ year), having more credit cards, being atheist or agnostic rather than protestant, and having a more pro-debt attitude.
Dwyer, McCloud, & Hodson [2]	Journal article (peer reviewed)	Quantitative, Cross-sectional	General population, Mixed, Ages 18 to 34	*N* = 3079	USA	Proportion of debt	Credit card debt has negative consequences on a sense of mastery and the level of self-esteem over time, possibly because financial stress adds up as young people age.
Grable & Joo [53]	Journal article (peer reviewed)	Quantitative, Cross-sectional	Students who participated in workshops on financial topics, Mixed, Approx. ages 18–23	*N* = 110	USA	Proportion of debt, Correlates of debt	Financial stress and negative financial behaviors (e.g., not making a spending plan, difficulty paying bills) were associated with credit card debt.
Ha [54]	Journal article (peer reviewed)	Quantitative, Cross-sectional	Students, Females, Ages 18–25	*N* = 257	Australia	Correlates of debt	Weekly income is significantly related to debt, marital status, educational level and age is not related to debt.
Haultain, Kemp, & Chernyshenko [55]	Journal article (peer reviewed)	Quantitative, Cross-sectional	Students Mixed, Ages 18	*N* = 125	New Zealand	Proportion of debt, Correlates of debt	Fear of debt was not associated with amount of debt and debt utility was positively associated with amount of debt
Henegar, Archuleta, Grable, Britt, Anderson, & Dale [56]	Journal article (peer reviewed)	Quantitative, Cross-sectional	General population, Mixed, Ages 18–38	*N* = 2618	USA	Proportion of debt	Gender (male), income, mother's financial impatience was associated with credit card debt.
Hoeve, Jak, Stams, & Meeus [34]	Journal article (peer reviewed)	Quantitative, Longitudinal	General population, Mixed, Ages 12–24 (at first debt measurement)	*N* = 1079	Netherlands	Debt-crime association	Financial problems were associated with self-reported delinquency. Effects of delinquency on financial problems were larger than effects of financial problems on delinquency.
Hogan, Bryant, & Overymyer-Day [57]	Journal article (peer reviewed)	Quantitative, Cross-sectional	Students, Mixed, Mean age = 20.5	*N* = 338	USA	Proportion of debt	Hours working, undesirable academics, shopping and perceived effect of work is associated with credit card balance; financial delinquency, hours studying, anxiety, drinking, gpa is not associated with credit card balance.
Houle [58]		Quantitative, Longitudinal	General population, Mixed, Ages 24–28	*N* = 4789	USA	Proportion of debt	Middle-income family is more strongly associated to debt than low- and high-income family; college-educated and high-income family is associated to low levels of debt; parental SES is more strongly associated with debt at private and high cost institutions.
Jessop, Herberts, & Solomon [8]	Journal article (peer reviewed)	Quantitative, Cross-sectional	Students, Mixed, Ages 18–50, (mean age 25)	*N* = 187	UK and Finland	Proportion of debt, Correlates of debt	Amount of debt was related to financial worries, cigarettes and alcohol use, physical, social and mental health. Amount of debt was not associated with hours worked, perceived control, role limitation due to physical and emotional problems and change in health.
Johnes [59]	Journal article (peer reviewed)	Quantitative, Cross-sectional	Students Mixed, Approx. ages 18–25	*N* = 1210	UK	Proportion of debt, Correlates of debt	Parent occupation, value of mandatory grant received, lower SES, being male, year of study (higher grade), having children, subject of study (subjects with relatively little immediate vocational relevance), being unmarried, depletion of savings was associated with the decision to take up student loans.
Joireman, Kees, & Sprott [60]	Journal article (peer reviewed)	Quantitative, Cross-sectional	Students, Mixed, Ages 18–35	*N* = 209	USA	Proportion of debt, Correlates of debt	Compulsive buying tendencies and importance to immediate consequences of actions were associated with amount of credit card debt. Compulsive buyers who focus on maximizing immediate consequences were at higher risk for substantial amounts of credit card debt.
Jones [61]	Journal article (peer reviewed)	Quantitative, Cross-sectional	Students, Mixed, Ages 18–30, (mean age 18.6)	*N* = 216	USA	Proportion of debt, Correlates of debt	Age (older students) and marital status (married) was associated with higher levels of debt. Knowledge of credit was not associated with debt.
Kerner, Weitekamp, Stelly, & Thomas [36]	Journal article (peer reviewed)	Quantitative, Longitudinal	Inmates Males Ages 20–30	*N* = 295	Germany	Debt-crime association	Minor delinquency and serious delinquency is associated with financial debt at age 25.
Lyons [62]	Journal article (peer reviewed)	Quantitative, Cross-sectional	Students, Mixed, Approx. Ages 18–35	*N* = 835	USA	Proportion of debt, Correlates of debt	Financially at risk students (e.g., large credit card debt, delinquent on payments) were more likely to be female, black, Hispanic, financially independent, receive financial aid, having debt other than credit card debt, having received credit cards by organizations other than a bank.
Moffitt, Caspi, Harrington, & Milne [30]	Journal article (peer reviewed)	Quantitative Longitudinal	General population Males, Age 26 (at follow-up)	*N* = 477	New Zealand	Debt-crime association	The recovery group, life-course persistent offenders and adolescent limited offenders had significantly more financial problems at age 26 than the unclassified comparison group.
Morra, Regehr, & Ginsburg [63]	Journal article (peer reviewed)	Quantitative, Cross-sectional	Students, Mixed, Approx. ages 23–26	*N* = 549	Canada	Correlates of debt	Financial stress was related to debt.
Nelissen, Van de Ven, & Stapel [37]	Journal article (peer reviewed)	Quantitative, Cross-sectional	Students, Mixed, Ages 13–19	*N* = 934	Netherlands	Proportion of debt, Correlates of debt	Status restoration was associated with adolescents' debt, particularly for those who had no other means to regain status (e.g., attractiveness or school achievement).
Noorda et al. [43]	Report	Qualitative, Cross-sectional	Homeless youth, Mixed, Ages 15–27	*N* = 73	Netherlands	Proportion of debt, Correlates of debt	Poor financial parenting, parental neglect, mental disability, parents with debt, poverty, compensation behavior, aggressive sales techniques were mentioned by participants and practitioners as explanations for debt.
Norvilitis & MacLean [15]	Journal article (peer reviewed)	Quantitative, Cross-sectional	Students, Mixed, Ages 19–26	*N* = 173	USA	Proportion of debt, Correlates of debt	Lack of belief that their parents would help them out if they found themselves in debt, lack of parents assistance in handling money, credit card problem use, credit card disinhibition (greater impulsivity when using credit card than other payment methods), (more) financial knowledge and poor financial well-being were related to debt, while parents' instruction, parents' worries about financial matters, parents' avoiding addressing financial matters were not.
Odgers, Moffitt, Broadbent, Dickson, Hancox, Harrington et al. [35]	Journal article (peer reviewed)	Quantitative, Longitudinal	General population Males Females Ages 7 at first measurement	*N* = 921	New Zealand	Debt-crime association	Life-course persistent offending is associated, and adolescent limited and child limited offending is not associated with financial problems at age 32 in females. Life-course persistent, adolescent limited, and child limited offending is associated with financial problems at age 32 in males.
Oosterbeek & Van den Broek [64]	Journal article (peer reviewed)	Quantitative, Cross-sectional	Students, Mixed, Average age 22	*N* = 5621	Netherlands	Proportion of debt, Correlates of debt	Borrowers were more likely to be older, male, university students, give themselves a higher probability to complete their studies, have a higher risk attitude, are less debt averse, spend more time on their study and are less likely to have a part-time job than non-borrowers. Borrowers' parents had higher incomes and are less debt averse.
Robb & Sharpe [40]	Journal article (peer reviewed)	Quantitative, Cross-sectional	Students, Mixed, Ages 18–30 (mean age 21)	*N* = 3884	USA	Proportion of debt, Correlates of debt	Financial independence, (more) financial knowledge, have lowly educated parents, having more than one credit card (other than obtained from a bank), being older was associated with debt.
Ross, Cleland & MacLeod [65]	Journal article (peer reviewed)	Quantitative, Cross-sectional	Students, Mixed, Approx. ages 18–22	*N* = 352	UK	Proportion of debt, Correlates of debt	Low SES and older students who report that their worries about money influences their study achievements, are more likely to have debt. Students with mental health problems are less likely to have debt. A temporary job and study results was not associated with debt.
Schwartz & Finnie [66]	Journal article (peer reviewed)	Quantitative, Cross-sectional	Students, Mixed, Mean age at follow-up 28	*N* = 80	Canada	Proportion of debt, Correlates of debt	Graduating with a student loan was not predicted by demand-side effects such as field of study and sex, but by supply-side factors (provincial student aid programs and different packaging of loans and grants). Problems with repayment of student loans were explained by low current earnings and fields likely to have low lifetime earnings. Females were more likely to have difficulty in repayment than males.
Shim, Xiao, Barber, & Lyon [9]	Journal article (peer reviewed)	Quantitative, Cross-sectional	Students, Mixed, Ages 18–24	*N* = 781	USA	Correlates of debt	Parents discuss financial issues and financial education was associated with financial knowledge, which in turn was associated with the intention to show positive financial behavior, which in turn was associated to higher financial well-being and lower levels of debt.
Siennick [31]	Dissertation	Quantitative, Cross-sectional	General population, Mixed, Ages 18–28	*N = *6581, *N = *14322	USA	Proportion of debt, Debt-crime association	Offenders receive more parental financial support and have higher incomes than non-offending counterparts, but they report more debt and economic hardship
Van Dam [32]	Dissertation	Quantitative, Cross-sectional	Former incarcerated males, Approx. ages 15–22 (mean age 19)	*N* = 57	Netherlands	Proportion of debt, Debt-crime association	Debt was associated with recidivism (frequency and seriousness of re-offenses).
Van Heijst & Verhagen [67]	Report	Descriptive, Cross-sectional	Students, Mixed, Approx. ages 16–23	*N* = 826	Netherlands	Proportion of debt, Correlates of debt	Being male, having a higher education, independent living, was associated with amount of debt.
Wang & Xiao [68]	Journal article (peer reviewed)	Quantitative Cross-sectional	Students, Mixed, Ages 18–30 (median age 21)	*N* = 272	USA	Proportion of debt, Correlates of debt	Compulsive buying, poor social support and budget constraint were related to credit card indebtedness. Mixed results were found for impulse buying. No relation was found between sex and parental income and credit card debt.
Zara & Farrington [33]	Journal article (peer reviewed)	Quantitative Longitudinal	High-risk sample, Males, Debt measured at Ages 16–18	*N* = 403	UK	Proportion of debt, Debt-crime association	Early starters (criminal activities before age 21) are more likely to have debt at age 16–18 compared to late starters (adult onset offenders).
Zhang & Kemp [7]	Journal article (peer reviewed)	Quantitative Cross-sectional	Students, Mixed, Age 17–68 (mean age 22)	*N* = 328	New Zealand	Proportion of debt, Correlates of debt	The amount of debt was not related to motivation towards education, academic performance happiness and attitude to debt.

### Coding of the study outcomes and characteristics

We retrieved the study results (test statistic and value) for proportions of debt (point prevalence), associations between correlates or risk factors and debt and associations between debt and offending. For each study result, we retrieved the sample size. We classified debt into the following types of debt: 1) general debt (borrowers, various types of loans), 2) credit card debt, 3) financial problems, 4) student loan, and 5) other specific type of debt, such as bank loan, and personal loan (loans from family and friends). If proportions of (types of) debt were reported, we coded whether the sample consisted of adolescents (ages 12–17), young adults (ages 18–30) or both adolescents and young adults (mixed age). Sex was coded as the percentage of females in the sample (0–100%), and we coded ethnicity as the percentage of ethnic minorities in the sample (non-Caucasian or nonindigenous subjects; 0–100%). In addition, we coded the number of items used to measure debt as an indicator of study quality (Number of debt items; 1–15). Further, publication year and the year of data collection were coded in order to be able to examine whether the level of debt has increased over the years. Finally, we coded the sample type (general population, students, high risk) and the continent where the data had been collected (North America, Europe, Australia).

For the meta-analyses of the correlates of debt we coded study result and sample size. We classified each correlate or risk factor of debt into one of the following domains: demographic, individual, family, peer, and financial. Further, we coded type of debt (general debt, credit card debt, financial problems) for each analysis. Studies that examined correlates of other types of debt, such as student loan, were not found.

Next, we retrieved study results and sample sizes of the association between debt and crime. Again, we coded type of debt (general debt, financial problems, specific types of debt, and specific financial problems). We also retrieved data on study design (cross-sectional, longitudinal), and coded whether debt and crime were measured simultaneously or whether debt was measured longitudinally before crime or vice versa. Further, we retrieved data on age group (adolescents, young adults), gender (males, females, mixed), and type of measure of criminal behavior (e.g., offenders vs. nonoffenders, delinquency trajectory, recidivism, severity).

### Analysis

For each study result an effect size was calculated. Proportions of debt (*ESp*; the number of subjects reporting debt divided by the total sample size) were transformed into logits (Lipsey & Wilson, 2001) for analysis and then, for presentation, transformed back into proportions. Further, we used the formulas of Mullen [21] and Lipsey and Wilson [22] to transform test statistics concerning the association between risk factors or correlates and debt and the association between debt and delinquency (e.g., χ^2^, *F*, *p*) into correlation *r* (*ESr*). If studies only reported that an association was significant or not, we applied conservative estimation procedures, meaning that we assigned a *p*-value of .50 if a non-significant effect was reported and a *p*-value of 0.05 for significant associations (Mullen, 1989). Each correlation was transformed into a Fisher's *Z* before combined effect sizes were calculated (Lipsey & Wilson, 2001; Mullen, 1989).

We conducted meta-analyses for each type of debt, that is, we computed mean proportions for general debt, credit card debt, financial problems and student loan, weighted by the inverse variance of the logit of *ESp* [22]. For the calculation of combined effect sizes and the moderator analyses, we used the SPSS macros of Lipsey and Wilson [22]. Given that most effect sizes were heterogeneous, we used random effects models. This method is rather conservative and has the advantage of allowing the results to generalize to studies that are not in the meta-analysis [22], [23].

For the meta-analyses on the association between risk factors or correlates and debt, we computed mean effect sizes weighted for the inverse variance for each correlate of debt. Next, we combined the dependent effect sizes (i.e., effect sizes within the same study) within the domains of correlates into a mean effect size before we computed mean effect sizes for each domain of correlates (demographic, individual, family, peer, and financial correlates of debt). Next, in order to examine potential differences between domains of correlates, we used a multilevel random effects model [24], [25] to conduct moderator analysis, relating domain to effect size. We used the program MLwiN for conducting multilevel analysis and used an adapted set up, described by Hox [24], to make our models suitable for meta-analysis. A multilevel random effects model accounts for the hierarchical structure of the data, in which the effect sizes or study results (the lowest level) are nested within studies (the highest level). In multilevel research, a random-effects model is often used, which can be extended by including moderators. Iterative maximum likelihood procedures are applied to estimate unknown parameters. The intercept only model (without moderators) is equivalent to the random-effects model of Hedges and Olkin [26]. In the complete model covariates can be added to test for potential moderators.

Given that we only found eight manuscripts that reported on only 39 analyses of the debt-crime association and that study characteristics were fairly different, we did not compute mean effect sizes, but instead chose to present the effect sizes (*ESr*) for each analysis (Table 2).

**Table 2:** Associations between Debt and Crime.

Study	Gender	Debt	Crime	*N*	*ESr*
Debt and Crime measured simultaneously					
General debt					
Zara and Farrington [33]	Males	debt	Late starters vs nonoffenders^c^	263	−.21
Zara and Farrington [33]	Males	debt	Offending < age 32 vs nonoffender^c^	403	.11
Zara and Farrington [33]	Males	debt	Early starters vs nonoffenders^c^	352	.19*
Van Dam [32]	Males	debt	Severity of recidivism^a^	57	.28*
Van Dam [32]	Males	debt	Recidivism^a^	42	.32*
Financial problems					
Blom et al. [29]	Mixed	Financial problems	Delinquency^a^	1,671	.21***
Hoeve et al. [34]	Mixed	Financial problems T1	Delinquency^a^ T1	1,258	.23***
Hoeve et al. [34]	Mixed	Financial problems T2	Delinquency^a^ T2	1,258	.25***
Hoeve et al. [34]	Mixed	Financial problems T3	Delinquency^a^ T3	1,258	.38***
Specific types of debt					
Siennick [31] – NLSY79 study	Mixed	student loan	Offenders vs nonoffenders^a^	1,902	−.04
Siennick [31] – Add Health study	Mixed	student loans	Offenders vs nonoffenders^a^	6,320	.00
Siennick [31] – NLSY79 study	Mixed	auto loan	Offenders vs nonoffenders^a^	1,902	.01
Siennick [31] – Add Health study	Mixed	credit card debt	Offenders vs nonoffenders^a^	6,320	.06**
Siennick [31] – NLSY79 study	Mixed	consumer debt	Offenders vs nonoffenders^a^	1,902	.07+
Siennick [31] – NLSY79 study	Mixed	personal loan	Offenders vs nonoffenders^a^	1,902	.27**
Specific types of financial problems					
Siennick [31] – Add Health study	Mixed	could not afford dentist	Offenders vs nonoffenders^a^	6,320	.08**
Siennick [31] – Add Health study	Mixed	could not afford doctor	Offenders vs nonoffenders^a^	6,320	.13***
Siennick [31] – Add Health study	Mixed	could not pay utility bills	Offenders vs nonoffenders^a^	6,320	.14***
Siennick [31] – Add Health study	Mixed	could not pay rent	Offenders vs nonoffenders^a^	6,320	.15***
Siennick [31] – Add Health study	Mixed	went without phone service	Offenders vs nonoffenders^a^	6,320	.16***
Siennick [31] – Add Health study	Mixed	evicted for nonpayment of rent	Offenders vs nonoffenders^a^	6,320	.21***
Siennick [31] – Add Health study	Mixed	utilities shut off for nonpayment	Offenders vs nonoffenders^a^	6,320	.23***
Debt measured before Crime					
Hoeve et al. [34]	Mixed	Financial problems T1	Delinquency^a^ T3	1,258	.08
Hoeve et al. [34]	Mixed	Financial problems T1	Delinquency^a^ T2	1,258	.08
Hoeve et al. [34]	Mixed	Financial problems T2	Delinquency^a^ T3	1,258	.14*
Crime measured before Debt					
General debt					
Kerner et al. [36]	Males	Financial debt age 25	Minor delinquency vs Nondel	218	.25***
Kerner et al. [36]	Males	Financial debt age 25	Serious delinquency vs Nondel	238	.48***
Financial problems					
Odgers et al. [35] – Dunedin Study	Females	Financial problems (age 32)	Child limited (vs Low)	374	.07
Odgers et al. [35] – Dunedin Study	Females	Financial problems (age 32)	AL path (vs Low)	361	.18
Hoeve et al. [34]	Mixed	Financial problems T2	Delinquency^a^ T1	1,258	.22***
Moffitt et al. [30] – Dunedin Study	Males	Financial problems	Recovery group (vs unclassified)^b^	272	.25***
Hoeve et al. [34]	Mixed	Financial problems T3	Delinquency^a^ T1	1,258	.25***
Moffitt et al. [30] – Dunedin Study	Males	Financial problems	AL path (vs unclassified)^b^	352	.27***
Odgers et al. [35] – Dunedin Study	Males	Financial problems (age 32)	AL path (vs Low)	352	.27*
Moffitt et al. [30] – Dunedin Study	Males	Financial problems	LCP path (vs unclassified)^b^	275	.29***
Hoeve et al. [34]	Mixed	Financial problems T3	Delinquency^a^ T2	1,258	.29***
Odgers et al. [35] – Dunedin Study	Males	Financial problems (age 32)	Child limited (vs Low)	377	.35*
Odgers et al. [35] – Dunedin Study	Females	Financial problems (age 32)	LCP path (vs Low)	331	.35*
Odgers et al. [35] – Dunedin Study	Males	Financial problems (age 32)	LCP path (vs Low)	302	.49*

*Note*. ^a^Self-reported; ^b^parent-, teacher- and self-reported; ^c^self-reported delinquency and convictions. *N* = number of participants; *ESr* = mean effect size correlation; AL = adolescence limited offenders; LCP = life-course persistent offenders; Recovery = extreme antisocial behavior in childhood but not in adolescence; Unclassified = not in AL, LCP, Recovery or Abstainer group; T1 = ages 12–24; T2 = ages 15–27; T3 = ages 18–30.

+ *p*<.10;

*p<.05;

**p<.01;

***p<.001.

## Results

### Description of studies

The total sample consisted of 36 manuscripts reporting on 32 independent samples. Findings on a total of 60,513 subjects were reported. Table 1 presents a description of the included studies. Studies were relatively recent; the oldest study was published in 1994, the most recent in 2014. The sample sizes were quite varied, ranging from 57 to 14,322. The designs were mostly cross-sectional (27 studies). Only six studies were longitudinal. The data included samples of only males (3 samples), only females (1 sample) or both males and females (28 samples). The majority of the studies concentrated on young adults (26 studies), whereas only three studies focused exclusively on adolescents (under age 18) and four studies had both adolescents and young adults in their sample. Furthermore, studies most often focused on students (21 studies), only six studies recruited participants from the general population. Finally, five studies investigated debt in deviant samples, including former detainees, homeless youth and high risk youth. Full data of individual studies are presented in Table S1 (proportions of debt), Table S2 (correlates of debt), and Table 2 (associations between debt and crime).

Of the 36 manuscripts, 23 reported on 36 proportions of (types of) debt or financial problems, 16 manuscripts reported on 123 analyses regarding correlates of debt and 8 manuscripts reported on 39 analyses concerning the association between debt and criminal behavior. Of the 36 proportions, 31 independent proportions of general debt, credit card debt, financial problems and student loan were used in the meta-analyses. The remaining proportions concerned very specific types of debt (e.g., auto loan) and because only one effect size was available for each of these types of debt, these proportions were not included in the meta-analyses. Of the 123 analyses regarding correlates of debt 114 were examined; only 9 analyses reported on financial problems (1 manuscript [27]) and we did not merge these effect sizes with either the general debt or credit card debt category.

### Prevalence of debt

Table 3 presents the overall mean proportion of general debt, credit card debt, financial problems and student loan. About half of the young people had some debt (*ESp* = .49), over a third had credit card debt (*ESp* = .36), a fifth had financial problems (*ESp* = .22) and over 40% had a student loan (*ESp* = .43). The prevalence of debt varied with age group, continent (see Table 3), and with sex, ethnic background and year of publication (see Table 4). For example, whereas over half of the young adults had debt (*ESp* = .56), only a quarter of the adolescents reported some debt (*ESp* = .24). Likewise, almost a third of the young adults (*ESp* = .29) versus 7% of the adolescents reported financial problems (*ESp* = .07; Table 3). Further, samples with more females were found to report more financial problems (β = .79, *Z* = 2.3, *p*<.05; see Table 4) and student loan (β = .98, *Z* = 5.3, *p*<.001). Samples with relatively many nonindigenous or noncaucasian participants reported more general debt (β = .85, *Z* = 3.5, *p*<.001). More recent publication years (β = .65, *Z* = 2.2, *p*<.05), but not years of data collection, were associated with higher proportions of credit card debt, and the association between year in which the data was collected and proportion of general debt (β = .39, *Z* = 1.9, *p*<.06) and financial problems (β = −.52, *Z* = 1.7, *p*<.06) was marginally significant and inconsistent (positive for general debt and negative for financial problems; Table 4). Given that at least 10 independent effect sizes were included in the meta-analysis on general debt, we inspected a funnel plot for these effect sizes. The funnel plot was roughly symmetrical (plot available on request).

**Table 3:** Results for the Overall Mean Proportion of Debt and Discrete Moderators by Type of Debt.

	General debt				Credit card debt				Financial problems				Student loan			
Category	*k*	*N*	*ESp*	*Q*	*k*	*N*	*ESp*	*Q*	*k*	*N*	*ESp*	*Q*	*k*	*N*	*ESp*	*Q*
Overall	11	15,366	.49	749.3***	7	22,043	.36	376.0***	5	17,691	.22	444.0***	5	20,762	.43	393.6***
Age group				8.8*				-				39.8***				-
Adolescents	2	1,337	.24		0	0	-		1	2,116	.07		0	0	-	
Young adults	6	13,073	.56		7	22,043	.36		4	15,575	.29		5	20,762	.43	
Mixed	3	956	.51		0	0	-		0	0	-		0	0	-	
Sample type				0.2				0.1				0.5				13.7***
General sample	1	6,581	.53		2	17,171	.34		2	16,438	.16		2	19,138	.26	
Students	7	8,252	.49		5	4,872	.37		3	1,253	.27		3	1,624	.56	
High risk youths	3	533	.46		0	0			0	0	-		0	0	-	
Continent				32.6***				3.3+				39.8***				11.2***
North America	2	6,797	.52		6	21,709	.39		4	15,575	.29		3	19,218	.31	
Europe	8	8,241	.40		1	334	.22		1	2,116	.07		2	1,544	.60	
Australia	1	328	.90		0	0	-		0	0	-		0	0	-	

*Note*. *k* = number of analyses, *N* = number of participants, *ESp* = mean effect size proportion *p*, *Q* = homogeneity statistic.

+ *p*<.10;

*p<.05;

**p<.01;

***p<.001.

**Table 4:** Results of Regression Analyses for Continuous Moderators to Predict Proportion of Debt.

	General debt				Credit card debt				Financial problems				Student loan			
	*k*	*N*	*Z*	Beta	*k*	*N*	*Z*	Beta	*k*	*N*	*Z*	Beta	*k*	*N*	*Z*	Beta
% Females	11	15,366	0.5	.11	7	22,043	1.3	.35	5	17,691	2.3*	.79	4	19,552	5.3***	.98
% Ethnic minorities	5	8,099	3.5***	.85	5	21,500	1.6	.40	4	17,611	0.9	.46	2	19,138	-	-
Year of publication	11	15,366	−0.1	−.03	6	19,194	2.2*	.65	5	17,691	−0.9	−.44	5	15,946	0.2	.15
Year data collection	11	15,366	1.9+	.39	6	19,194	0.9	.42	5	17,691	−1.7+	−.52	5	15,946	0.4	.35
Number of debt items	9	14,890	−2.0+	−.43	7	22,043	−1.7+	−.40	5	17,691	0.4	.24	5	20,762	-	-

*Note*. *k* = number of analyses, *N* = number of participants.

+ *p*<.06;

*p<.05;

**p<.01;

***p<.001.

### Risk factors and correlates of debt

Studies reported on various correlates of financial problems of indebtness (see Table 5). According to the criteria of Cohen [28], who proposed that correlations of .10, .25, and .40, are small, medium and large effect sizes respectively, the correlations range from small to large. We found a range of nonsignificant small effects (e.g., *ESr* = .02 for hours worked or *ESr* = −.01 happiness) to significant large effects (e.g., *ESr* = .37 for perceived control toward financial management, *ESr* = .39 for parents attitude to debt, and *ESr* = .55 for financial stress). Consistent with our moderator analyses of the proportion of debt, older age was associated with higher levels of debt (*ESr* = .12, *Z* = 12.85, *p*<.001). Likewise, more advanced students reported relatively more debt (study year, *ESr* = .31, *Z* = 14.24, *p*<.001). Further, ethnicity was associated with debt (*ESr* = .16, *Z* = 11.99, *p*<.001 for general debt; *ESr* = .23, *Z* = 2.43, *p*<.05 for credit card debt). Surprisingly, higher income was associated with higher debt (*ESr* = .26, *Z* = 11.36, *p*<.001) and higher credit card debt (*ESr* = .36, *Z* = 6.06, *p*<.001).

**Table 5:** Mean Effect Sizes for (Domains of) Correlates of General Debt and Credit Card Debt.

	General debt				Credit card debt			
Demographic	*k*	*N*	*ESr*	*Z*	*k*	*N*	*ESr*	*Z*
Age	4	11,249	.12	12.85***	1	257	.00	0.00
Ethnic minority	2	2,742	.16	11.99***	1	110	.23	2.43*
Sex (Female)	7	13,464	.02	1.91*	1	398	.05	0.97
SES	2	5,638	−.15	−11.40***				
Region (US south)	1	5,304	.06	4.37***				
Urbanization	1	5,304	.03	1.97*				
Marital status	1	180	.12	1.60	1	257	.00	0.00
Holds part-time job	1	5,621	.02	1.80+				
Hours worked	3	6,142	.02	1.87+				
Income	3	1879	.26	11.36***	1	257	.36	6.06***
Earnings after graduation	1	5,621	.00	0.48				
Study hours	1	5,621	.03	2.25*				
Study year	4	1,992	.31	14.24***	1	257	.00	0.00
Financial education	1	781	.03	0.84				
State school (vs. private school)					1	399	.15	3.10**
Overall	9	14,104	.08	9.24***	3	766	.12	3.27***
Individual	*k*	*N*	*ESr*	*Z*	*k*	*N*	*ESr*	*Z*
Risk attitude	1	5,621	.11	8.58***				
Mental health	2	521	−.11	−2.59***				
Physical health	1	187	−.15	−5.75***				
Happiness	1	328	−.01	−0.18				
Self-esteem	3	7,003	−.29	−24.53***				
Social functioning	1	187	−.28	−3.90***				
School performance	3	5,324	−.05	−3.90***				
Motivation towards study (intrinsic)	1	328	.16	3.98***				
Probability to find suitable job	1	5,621	.02	1.72+				
Locus of control (external)	2	5,491	.23	17.42***				
Seek social support					1	272	.00	0.00
Delay of gratification					1	173	−.10	−1.31
Take into account consequences of behavior					1	209	−.17	−2.46*
Overall	7	12,706	.12	13.45***	3	654	.08	2.06*
Family	*k*	*N*	*ESr*	*Z*	*k*	*N*	*ESr*	*Z*
Lived with parents	1	5,304	−.11	−7.85***				
Parental income	2	5,621	−.08	−6.38***	1	272	.00	0.00
Change in family income	1	5,304	.20	14.90***				
Parents attitude to debt (pro-debt)	1	4,764	.39	28.50***				
Willingness to meet parental expectations (towards positive financial behaviors)	1	781	.01	0.28				
Parent talking about finances	1	781	−.11	−3.08**	1	173	−.02	−0.26
Parental financial support					1	173	−.17	−3.09**
Parent bailout					1	173	−.29	−3.89***
Parent worries					1	173	.04	0.52
Overall	3	10,887	.19	20.27***	2	445	.05	1.13
Peer	*k*	*N*	*ESr*	*Z*				
Social comparison tendency	1	918	−.01	−0.30				
Status concern	1	918	.08	4.20***				
Overall	1	918	.06	3.49***				
Financial	*k*	*N*	*ESr*	*Z*	*k*	*N*	*ESr*	*Z*
Attitude towards debt (pro-debt)	4	6,992	.34	29.84***				
Discount rate (valuation of current vs. future consumption)	1	5,621	.17	12.87***				
Rated extent to which debt affects happiness	1	328	−.02	−0.36				
Perceived control toward financial management	1	781	−.37	−10.83***				
Attitudes towards financial management	1	781	−.01	−0.28				
Financial management intention	1	781	−.18	−5.08***				
Financial anxiety	1	180	.27	3.68**				
Financial wellbeing (vs. stress)	5	2,031	−.29	−13.18***	1	173	−.55	−8.06***
Financial knowledge	2	481	.07	2.30*	1	173	.20	2.64**
Budget constraint					1	272	−.10	−1.65
Compulsive buying					1	272	.10	2.33*
Overall	9	9,624	.23	22.59***	3	654	.27	7.02***

*Note*. *k* = number of analyses, *N* = number of participants, *ESr* = (mean) effect size correlation *r*.

+ *p*<.10;

*p<.05;

**p<.01;

***p<.001.

In the individual domain of correlates, we found medium to large effect sizes for locus of control (*ESr* = .23, *Z* = 17.42, *p*<.001), social functioning (*ESr* = −.28, *Z* = −3.9, *p*<.001), and self-esteem (*ESr* = −.29, *Z* = −24.53, *p*<.001), indicating that those with an external locus of control, poor social functioning and low self-esteem reported higher amounts of debt. Studies reported small but significant effects for risk attitude (*ESr* = .11, *Z* = 8.58, *p*<.001), and mental (*ESr* = −.11, *Z* = −2.59, *p*<.001) and physical health (*ESr* = −.15, *Z* = −5.75, *p*<.001). Further, those who take the consequences of their behavior into account were less likely to have credit card debt (*ESr* = −.17, *Z* = −2.46, *p*<.05). Students with relatively lower levels of school performance (*ESr* = −.05, *Z* = −3.90, *p*<.001), but higher intrinsic motivation towards their studies (*ESr* = .16, *Z* = 3.98, *p*<.001) were more likely to report higher levels of debt.

Several family characteristics were found to be associated with indebtness. The strongest effect size was found for parents attitude to debt (*ESr* = .39, *Z* = 28.5, *p*<.001), indicating that those whose parents' attitude was in favor of debt were more likely to have debt. Further, young people who reported that parents would not bail them out if necessary (*ESr* = −.29, *Z* = 3.89, *p*<.001), were more likely to have credit card debt. Those whose parents had relatively low incomes were more likely to report debt (*ESr* = −.08, *Z* = −6.38, *p*<.001).

With regard to peer factors, only status concern was found to be associated with debt (*ESr* = .08, *Z* = 4.20, *p*<.001). Particularly, young adults with high levels of public self-consciousness, who were concerned about other people's opinions about them and who worried about what kind of impression they made on others, were more likely to report debt.

The strongest effect sizes were found in the domain of financial correlates of debt (*ESr* = .23, *Z* = 22.59, *p*<.001 for general debt; *ESr* = .27, *Z* = 7.02, *p*<.001for credit card debt). Financial stress was most strongly associated with debt, particularly with credit card debt (*ESr* = −.55, *Z* = 8.06, *p*<.001). Further, strong effects were found for attitude towards debt (*ESr* = .34, *Z* = 29.84, *p*<.001). Those with a pro-debt attitude were more likely to report higher amounts of debt.

We tested whether mean effect sizes of individual, family, peer and financial domains of correlates were significantly different from the demographic domain (the reference category). In order to do this, we conducted multilevel meta-analysis on all effect sizes concerning correlates of debt (*k* = 114; general and credit card debt). Mean effect sizes of individual (β = .06, *SD* = .01, *Z* = 10.3, *p*<.001), family (β = .13, *SD* = .01, *Z* = 18.9, *p*<.001), and financial (β = .19, *SD* = .01, *Z* = 23.1, *p*<.001) correlates of debt were significantly larger than the mean effect size of demographic correlates (intercept β = .06, *SD* = .02, *Z* = 2.5, *p*<.01). We found that the mean effect size of the peer domain was significantly smaller than the demographic domain (β = −.05, *SD* = .02, *Z* = −2.3, *p*<.05). The model predicted effect size significantly better than the model without domains of correlates (Δχ^2^ = 715.5, *p*<.001). We inspected the shape of funnel plots for categories of effect sizes which consisted of at least 10 independent effect sizes (demographic, individual, and financial). The plot of the demographic correlates was somewhat skewed to the right indicating possible publication bias, studies reporting relatively small effect sizes may not have been included in this meta-analysis. The remaining funnel plots were roughly symmetrical (plots available on request). We also calculated fail-safe numbers to estimate the number of unretrieved studies averaging null results needed to bring the overall medium effect sizes (financial correlates) at a small level. We found fail-safe numbers of 14 (9[.25/.10–1]) and 5 (3[.27/.10–1]). Given that a thorough search has been undertaken, searching for both published and unpublished studies, it is unlikely that with 9 and 3 studies included, 14 and 5 studies respectively have remained unfound.

### The association between debt and delinquency

Eight studies [29]–[36] focused on the association between financial debt and crime. Overall, the vast majority of effect sizes indicated that debt is significantly associated with criminal behavior in adolescents and young adults (Table 2). From the table it becomes clear that when considering studies in which debt and crime were measured simultaneously, the strongest effect size was found for young adults; it appears that the association between financial problems and delinquency becomes stronger with age (*ESr* ranges from .23 for ages 12–24 to .38 for ages 18–30). Also, relatively strong associations were found between debt and recidivism (*ESr* = .32, *p*<.001), suggesting that those juvenile offenders who recidivate are more likely to have debt.

Considering specific types of debt, it was found that offenders compared to nonoffenders were particularly more likely to have personal or unofficial debt (*ESr* = .27, *p*<.001), that is, debt from family and peers (Table 2). Effect sizes for student and auto loan were nonsignificant, indicating that these types of debt are not assocatiated with criminal behavior in young people. Concerning specific types of financial problems, offenders were found to be likely to have all kinds of financial problems than nonoffenders, not being able to pay rent (*ESr* = .21, *p*<.001) or utility bills (*ESr* = .23, *p*<.001) in particular.

Several studies examined the debt-delinquency link longitudinally, with most studies measuring crime before debt than vice versa. From Table 2 it becomes clear that relatively stronger associations between debt and crime were found when crime was measured before debt than the other way around. Effect sizes up to .49 were found when crime was measured before debt, while effect sizes were nonsignificant or small when debt was measured before crime. Further, from the longitudinal analyses (Crime measured before Debt) it becomes clear that associations between serious and persistent offending and debt or financial problems were large in magnitude. For example, serious delinquents were more likely to have financial debt later in life at age 25 (*ESr* = .48, *p*<.001). Likewise, life-course persistent offenders were more likely to have financial problems at age 32 compared to those with low rates of delinquency (*ESr* = .49, *p*<.001). Although effect sizes for females (*ESr*s ranges from .07 to .35) were overall somewhat smaller than those of males (*ESr*s ranges from .25 to .49), associations between female life-course persistent offending and future financial problems were strong as well (*ESr* = .35, *p*<.01).

## Discussion

The present systematic review and meta-analysis summarized and integrated previous findings on 1) the prevalence of financial debt among adolescents and young adults, 2) correlates and risk factors of debt, and 3) the association between debt and crime. We found 36 manuscripts that reported on at least one of these three topics. Findings revealed that the prevalence of debt is substantial among young people. About half reported to have at least some debt (49%) and almost a quarter had one or another financial problem (22%). These findings confirm earlier research [1]–[3]. However, evidence from the present meta-analyses suggesting that financial debt has increased in recent years is weak. Older participants and ethnic minorities were found to have higher levels of debt than younger and indigenous counterparts. Females had more financial problems and higher student loans. We found considerably strong associations between debt and crime. Particularly, large effect sizes were found for serious and persistent crime in young people and later (young adult) debt or financial problems.

### Risk factors and correlates of debt

Given that correlates were found in various domains, it can be concluded that a variety of factors explain indebtness in young people, including demographic, individual, family, peer, societal and more proximal financial factors (e.g., positive attitude towards debt, financial stress). On average, the largest effects were found for financial factors. Youths who experience stress related to their finances, who find it difficult to control finances (e.g., spending within budget or saving money) and those who find it not problematic to have debt (pro-debt attitude) are more likely to have debt. Surprisingly, this meta-analysis on correlates of debt showed that higher levels of financial knowledge is associated with higher levels of debt, regardless how knowledge was measured (assessment or self-evaluation). One of the studies that could not be included in our meta-analysis because the researchers conducted multivariate analyses [39] showed that *poor* financial knowledge was associated with debt, as expected, while another multivariate study showed that *more* financial knowledge was associated with debt [40]. Specific financial management skills may be more effective in explaining no or low levels of debt than financial knowledge. For example, in one study [15], more parental instructions on finances and assistance in money handling was associated with *less* debt.

We also found several large effect sizes in the individual domain, indicating that those youths who report low levels of self-esteem, poor social functioning and high levels of external locus of control are at risk for financial debt. An alternative explanation could be that financial debt results in poor self-esteem and an external locus of control, but one of the included study measured these correlates at an earlier time point than financial debt.

Further, parents seem to increase the likelihood of their offspring's debt: if parents talk about finances and provide financial support, youths are less likely to report debt. In addition, when parents have a pro-debt attitude, youths are more likely to have debt. Youths may have adopted their parents' pro-debt attitude as we also found associations between youths' pro-debt attitudes and debt. Small effects of peers were found. Evidence was found that status concern, particularly status restoration, was associated with adolescent debt [37]. This effect was moderated by attractiveness and school performance: for attractive and bright students no significant association was found, whereas for those who had low grades and low ratings of attractiveness, status restoration was significantly associated with debt. These negatively evaluated youths may have bought stuff in order to restore their self-integrity [37].

A qualitative study [38], comparing findings from the UK and Ireland, found evidence to suggest that societal and cultural factors affect debt in students, that is, a credit-oriented society was shown to affect student debt. Students referred to a trend of increasing debt in their country, and their perceptions of the environment indicated that indebtness had become normalized. Further, the easiness to obtain credit cards and marketing approaches of institutions were associated with increased levels of student debt [38]. Overall, the present meta-analysis' findings suggest that debt of young people is influenced by a range of factors in different domains, varying from proximal financial management factors to distal societal and cultural factors.

### Debt and crime

Expectably, we found that debt and financial problems are associated with crime. The strength of the association between delinquency and debt seems to vary with type of offender. Particularly, serious and life-course persistent offenders (*ESr* = .48–.49) were much more likely to have debt compared to counterparts who do not commit crimes or only engage in nonserious delinquency. Childhood-onset or life-course persistent offenders typically follow a delinquent path that starts in the early teens, entails many delinquent acts, and persists far into adulthood [41]. Overt aggressive and more serious offenses are more common in early-onset delinquents. These offenders are furthermore characterized by problems in their childhood, such as poor family functioning and neuro-cognitive impairment [42]. Thus, although general criminal behavior is associated with debt and financial problems, it seems that serious persistent offenders in particular are more likely to have debt.

Findings that debt is associated with criminal behaviour are consistent with assumptions of strain theorists [11], [12], who argue that people experiencing financial or economic strain are more likely to engage in crime. However, we found longitudinal associations between crime and debt suggesting that delinquent adolescents and young adults are more likely to develop financial problems, maybe due to personal or unofficial debt, that is, debt from family and peers [31], [43] or financial penalties. With regard to the direction of the effect, delinquent behaviour seems to be a risk factor for having problematic debts. The paths from criminal behaviour to debt and financial problems were stronger than the other way around [34]. Delinquent youth may follow different routes to financial debts. For example, life course persistent offenders may have debts because their early onset offending initiates a chain of cumulative problems in various domains, while adolescent limited offenders may have debts due to their relatively high impulsiveness [30].

Interestingly, focusing on specific types of debt it was found that offenders were more likely to have personal loans, which are loans from family or peers or other relatives. This finding was shown in a quantitative study [31] as well as in a qualitative study [43]. Particularly, in a sample of homeless youths, having so-called informal or street debt proved to be a significant problem. This type of debt originates from borrowing money from peers and is connected with illegal activities, such as fraud, drug dealing, theft and violence. This category of debt is more problematic than formal debt, because youths cannot pay them off in a formal way [43]. Thus, particularly in vulnerable youths, debt and delinquency can be closely intertwined. Given that this type of debt is not registered, it may be more difficult to trace and to identify financial problems related to this type of debt.

Research has found that poverty in families and neighborhoods and low family SES is associated with criminal behavior [44]. In the present review we found that low SES is associated with financial debt, too. Low SES and possibly other shared risk factors may explain the association between financial debt and crime. However, Siennick [31], examining the association between debt and crime in two large US samples, found that financial problems were greater in offenders than in non-offenders, regardless the resources of the families of origin. Thus, even offenders with relatively wealthy familial backgrounds are more likely to have financial debt. In addition, offenders generally had higher incomes than non-offenders [31]. This suggests that needs and desires may be higher in offenders, net of their financial resources, and that the discrepancy between needs and resources explains their criminal behavior.

### Limitations and research gaps

From our review it became apparent that several research gaps exist. Most importantly, longitudinal studies were limited. In order to increase knowledge on the etiology of debt and the direction of the association between debt and crime, longitudinal studies are needed. In order to develop effective interventions that target finances and debt in young offenders with the aim of preventing recidivism, there is a need for further research on financial debt in young people and associations with criminal behavior.

Further, studies on samples that specifically focused on males only or females only were limited. Although attention to criminal behavior in young females has been increasing in recent years [45], studies on debt in young women seem to be almost absent. Finally, studies on samples other than student samples were scarce. Particularly, studies on vulnerable youth, such as homeless youth and addicted youth, are needed. A review of Gupta and Derevensky [46] showed that pathological juvenile gamblers were more likely to have debt *and* engage in delinquency, but, to our knowledge, the relation between debt and delinquency in gamblers has not been investigated.

A substantial amount of young people (about half) are in debt and even almost a quarter have financial problems, and therefore this problem merits more attention of youth practitioners and policy makes. Given that the oldest study on debt in young people we found is as recent as 1994, further research is warranted.

### Implications for policy and practice

The current investigation has several implications for policy and practice. Strong correlations between serious and persistent offending and debt were found. The practical importance of a correlation can be shown in a Binomial Effect Size Display (BESD,[47]). For example, consider a group of 200 youngsters of which half of these youngsters are serious offenders and half are not. A correlation of .48 can be displayed as follows: 74 out of 100 serious offenders compared to only 26 out of 100 nonoffending youngsters are expected to have financial debts. Therefore, interventions and aftercare programs for delinquents should focus on dealing with debt and financial problems. Given that financial debt was associated with recidivism in post-incarcerated youths, targeting financial problems in these offenders effectively might reduce the risk of future offenses. The finding that those who engage in crime have relatively often personal loans, is of concern for practitioners who work with delinquent youths.

A few studies found some evidence that interventions that target financial problems are effective. For example, debt advise had decreased financial debt in adolescents and young adults after one year [48]. A recent review of financial interventions showed that financial education programs often are not evaluated and that studies that examined potential effects are of poor quality [49]. Some evidence was found that financial education is effective, though, and that effectiveness was associated with the youth's motivation for improvement of financial knowledge and skills [49].

Given that studies of debt in the present systematic review have shown that various factors are related to debt, programs should not only focus on financial knowledge and money management, but also on risk factors in other domains. A program solely focusing on financial knowledge may not result in decreasing youths' financial problems and debt, as our review found that higher levels of financial knowledge is related to more debt. Given that the strongest associations with debt were found for low self-esteem, a pro-debt attitude (of young people and their parents), perceived control towards financial management, poor social functioning, financial stress and external locus of control, interventions should target these issues.

Finally, the finding that having multiple credit cards is associated with higher debt offers a point of departure for theoretically founded policy measures. In addition, the finding that a credit-friendly society enhances debt in young people, as has been found by a qualitative study [38], suggests that policy that is aimed at reducing general debt in society and at altering perceptions that promote having debts might reduce debt in young people. It is important that future studies empirically test the effectiveness of such policy measures and interventions.

## Supporting Information

Figure S1 **Prisma 2009 Flow Diagram.**
(TIF)

Table S1 **Proportions of debt.**
(PDF)

Table S2 **Correlates of debt.**
(PDF)

Checklist S1 **Prisma 2009 Checklist.**
(PDF)

## References

[1] Betti G, Dourmashkin N, Rossi M, Yin YP (2007) Consumer over-indebtedness in the EU: Measurement and characteristics. Journal of Economic Studies 34: 136–156.

[2] Dwyer RE, McCloud L, Hodson R (2011) Youth debt, mastery, and self-esteem: Class-stratified effects of indebtedness on self-concept. Social Science Research 40: 727–741.

[3] Verhagen S, Van Heijst P, Jurrius K, Calkoen P, Koot E (2010) Rood staan is geen schuld: Hoe professionals geldproblemen bij jongeren kunnen voorkomen [Credit is not the same as debt: How professionals can prevent financial problems in youth]. Tijdschrift voor Sociale Vraagstukken jaargang 2010: 8–11.

[4] Draut T, Silva J (2004) Generation broke: The growth of debt among young Americans. New York: DEMOS.

[5] National Center of Education Statistics (2010) Digest of education statistics 2009. Washington DC: NCES. Available: www.nces.ed.gov/programs/digest. Accessed 25 July 2011.

[6] Billieux JL, Van der Linden M, Rochat L (2008) The role of impulsivity in actual and problematic use of the mobile phone. Applied Cognitive Psychology 22: 1195–1210.

[7] Zhang J, Kemp S (2009) The relationships between student debt and motivation, happiness, and academic achievement. New Zealand Journal of Psychology 38: 24–29.

[8] Jessop DC, Herberts C, Solomon L (2005) The impact of financial circumstances on student health. British Journal of Health Psychology 10: 421–439. 16238857 10.1348/135910705X25480

[9] Shim S, Xiao JJ, Barber BL, Lyons AC (2009) Pathways to life success: A conceptual model of financial well-being for young adults. Journal of Applied Developmental Psychology 30: 708–723.

[10] Farrington DP (2003) Developmental and life-course criminology: Key theoretical and empirical issues. Criminology 41: 221–255.

[11] Merton RK (1938) Social structure and anomie. American Sociological Review 3: 672–682.

[12] Agnew R (1985) A revised theory of delinquency. Social Forces 64: 151–167.

[13] Gottfredson MR, Hirschi T (1990) A general theory of crime. Palo Alto: Stanfort University Press.

[14] Jessor R (1991) Risk behavior in adolescence: A psychosocial framework for understanding and action. Journal of Adolescent Health 12: 597–605. 1799569 10.1016/1054-139x(91)90007-k

[15] Norvilitis JM, MacLean MG (2010) The role of parents in college students' financial behaviors and attitudes. Journal of Economic Psychology 31: 55–63.

[16] Palmer TS, Pinto MB, Parente DH (2001) College students' credit card debt and the role of parental involvement: Implications for public policy. Journal of Public Policy & Marketing 20: 105–113.

[17] Rosenthal R (1991) Meta-analytic procedures for social research. Newbury Park, CA: Sage.

[18] Van IJzendoorn MH (1998) Meta-analysis in early childhood education: Progress and problems. In: Spodek B, Saracho ON, Pellegrini AD, editors. Issues in early childhood educational research. Yearbook in early childhood education. New York: Teachers College. pp. 156–176.

[19] Rosenthal R (1979) The "file drawer problem" and tolerance for null results. Psychological Bulletin 86: 638–641.

[20] Rothstein HR (2008) Publication bias as a threat to the validity of meta-analytic results. Journal of Experimental Criminology 4: 61–81.

[21] Mullen B (1989) Advanced BASIC meta-analysis. New York: Erlbaum.

[22] Lipsey MW, Wilson DB (2001) Practical meta-analysis. Thousand Oaks: Sage.

[23] Rosenthal R (1995) Writing meta-analytic reviews. Psychological Bulletin 118: 183–192.

[24] Hox J (2002) Multilevel analysis: Techniques and applications. New York: Lawrence Erlbaum Associates.

[25] Van den Noortgate W, Onghena P (2003) Multilevel meta-analysis: A comparison with traditional meta-analytical procedures. Educational and Psychological Measurement 63: 765–790.

[26] Hedges LV, Olkin I (1985) Statistical methods for meta-analysis. New York: Academic Press.

[27] Crocker J, Luhtanen RK (2003) Level of self-esteem and contingencies of self-worth: Unique effects on academic, social, and financial problems in college students. Personality and Social Psychology Bulletin 29: 701–712. 15189626 10.1177/0146167203029006003

[28] Cohen J (1988) Statistical power analysis for the behavioral sciences. New York: Academic Press.

[29] Blom M, Weijters G, Van der Laan AM (2011) Problemen met geld en delinquent gedrag van adolescenten [Financial problems and delinquent behavior of adolescents] (factsheet 2011-1). Den Haag: WODC.

[30] Moffitt TE, Caspi A, Harrington H, Milne BJ (2002) Males on the life-course-persistent and adolescence-limited antisocial pathways: Follow-up at age 26 years. Development and Psychopathology 14: 179–207. 11893092 10.1017/s0954579402001104

[31] Siennick SE (2009) Three essays on criminals' indebtedness. Available: http://gradworks.umi.com/34/80/3480746.html. Accessed 24 September 2012.

[32] Van Dam C (2005) Juvenile criminal recidivism: Relations with personality and post release environmental risk and protective factors.

[33] Zara G, Farrington DP (2010) A longitudinal analysis of early risk factors for adult-onset offending: What predicts a delayed criminal career? Criminal Behaviour and Mental Health 20: 257–273. 20306484 10.1002/cbm.763

[34] Hoeve M, Jak S, Stams GJJM, Meeus WHJ (2014) Financial problems and delinquency in adolescents and young adults: A six-year three-wave study. *Crime & Delinquency, xx*, 1-22. Available: http://cad.sagepub.com/content/early/2014/06/23/0011128714541190. Accessed: 27 June 2014.

[35] Odgers CL, Moffit TE, Broadbent JM, Dickson N, Hancox RJ, et al (2008) Female and male antisocial trajectories: From childhood origins to adult outcomes. Development and Psychopathology 20: 673–716. 18423100 10.1017/S0954579408000333

[36] Kerner H, Weitekamp EGM, Stelly W, Thomas J (1997) Patterns of criminality and alcohol abuse: Results of the tuebingen criminal behaviour development study. Criminal Behaviour and Mental Health 7: 401–420.

[37] Nelissen RMA, Van de Ven N, Stapel D (2011) Status concerns and financial debts in adolescents. Social Influence 6: 39–56.

[38] O'Loughlin D, Szmigin I (2006) "I'll always be in debt": Irish and UK student behaviour in a credit led environment. Journal of Consumer Marketing 23: 335–343.

[39] Norvilitis JM, Merwin MM, Osberg TM, Roehling PV, Young P, et al (2006) Personality factors, money attitudes, financial knowledge, and credit-card debt in college students. Journal of Applied Social Psychology 36: 1395–1413.

[40] Robb CA, Sharpe DL (2009) Effect of personal financial knowledge on college students' credit card behavior. Journal of Financial Counseling and Planning 20: 25–43.

[41] Moffitt TE (1993) Adolescence-limited and life-course-persistent antisocial behavior: A developmental taxonomy. Psychological Review 100: 674–701. 8255953

[42] Moffitt TE, Caspi A (2001) Childhood predictors differentiate life-course-persistent and adolescence-limited antisocial pathways among males and females. Development and Psychopathology 13: 355–375. 11393651 10.1017/s0954579401002097

[43] Noorda J, Pehlivan T, Clement C, Ezzeroili L, Neijboer D, et al (2009) Risicojongeren, schulden en huisvestingsnood: Verkennend onderzoek naar omvang, aard en aanpak van schulden en huisvestingsproblemen onder risicojongeren [At-risk youth, debt and homelessness]. New York: Stichting Alexander/Noorda en Co.

[44] Hsieh CC, Pugh MD (1993) Poverty, income inequality, and violent crime: A meta-analysis of recent aggregate data studies. Criminal Justice Review 18: 182–202.

[45] Zahn MA (2009) The delinquent girl. New York: Temple University Press.

[46] Gupta R, Derevensky J (2000) Adolescents with gambling problems: From research to treatment. Journal of Gambling studies 16: 315–342. 14634318 10.1023/a:1009493200768

[47] McCartney K, Rosenthal R (2000) Effect size, practical importance, and social policy for children. Child Development 71: 173–180. 10836571 10.1111/1467-8624.00131

[48] Williams K, Sansom A (2007) Twelve months later: Does advice help? the impact of debt advice – advice agency clients study. Ministry of Justice Research Series 6/07.

[49] McCormick MH (2009) The effectiveness of youth financial education: A review of the literature. Journal of Financial Counseling and Planning 20: 70–83.

[50] Archuleta KL, Dale A, Spann SM (2013) College students and financial distress: Exploring debt, financial satisfaction, and financial anxiety. Journal of Financial Counseling and Planning 24: 50–62.

[51] Caputo RK (2012) Patterns and predictors of debt: A panel study, 1985–2008. Journal of Sociology & Social Welfare 39: 7–29.

[52] Davies E, Lea SEG (1995) Student attitudes to student debt. Journal of Economic Psychology 16: 663–679.

[53] Grable JE, Joo S (2006) Student racial differences in crerdit card debt and financial behaviors and stress. College Student Journal 40: 400–408.

[54] Ha H (2013) Credit card use and debt by female students. Youth Studies Australia 32: 57–71.

[55] Haultain S, Kemp S, Chernyshenko OS (2010) The structure of attitudes to student debt. Journal of Economic Psychology 31: 322–330.

[56] Henegar JM, Archuleta K, Grable J, Britt S, Anderson N, et al (2013) Credit card behavior as a function of impulsivity and mother's socialization factors. Journal of Financial Counseling and Planning 24: 37–49.

[57] Hogan E, Bryant SK, Overymyer-Day LE (2013) Relationships between college students' credit card debt, undesirable academic behaviors and cognitions, and academic performance. College Student Journal 47: 102–112.

[58] Houle JN (2014) Disparities in debt: Parents' socioeconomic resources and young adult student loan debt. Sociology of Education 87: 53–69.

[59] Johnes G (1994) The determinants of student loan take-up in the united kingdom. Applied Economics 26: 999–1005.

[60] Joireman J, Kees J, Sprott D (2010) Concern with immediate consequences magnifies the impact of compulsive buying tendencies on college students' credit card debt. Journal of Consumer Affairs 44: 155–178.

[61] Jones JE (2005) College students' knowledge and use of credit. Financial counseling and planning 16: 9–16.

[62] Lyons AC (2004) A profile of financially at-risk college students. The Journal of Consumer Affairs 38: 56–80.

[63] Morra DJ, Regehr G, Ginsburg S (2008) Anticipated debt and financial stress in medical students. Medical Teacher 30: 313–315. 18484459 10.1080/01421590801953000

[64] Oosterbeek H, Van den Broek A (2009) An empirical analysis of borrowing behaviour of higher education students in the Netherlands. Economics of Education Review 28: 170–177.

[65] Ross S, Cleland J, MacLeod MJ (2006) Stress, debt and undergraduate medical student performance. Medical Education 40: 584–589. 16700775 10.1111/j.1365-2929.2006.02448.x

[66] Schwartz S, Finnie R (2002) Student loans in Canada: An analysis of borrowing and repayment. Economics of Education Review 21: 497–512.

[67] Van Heijst P, Verhagen S (2009) Jongeren en schulden: De leerlingen van ROC midden Nederland in beeld [Youth and debt]. Utrecht, the Netherlands: SWP.

[68] Wang J, Xiao JJ (2009) Buying behavior, social support and credit card indebtedness of college students. International Journal of Consumer Studies 33: 2–10.

