# Supplementary figures and images for: A Systematic Review of Financial Debt in Adolescents and Young Adults: Prevalence, Correlates and Associations with Crime
Source: PLoS One. 2014 Aug 19;9(8):e104909. doi: 10.1371/journal.pone.0104909 (PMC4138212; doi:10.1371/journal.pone.0104909)

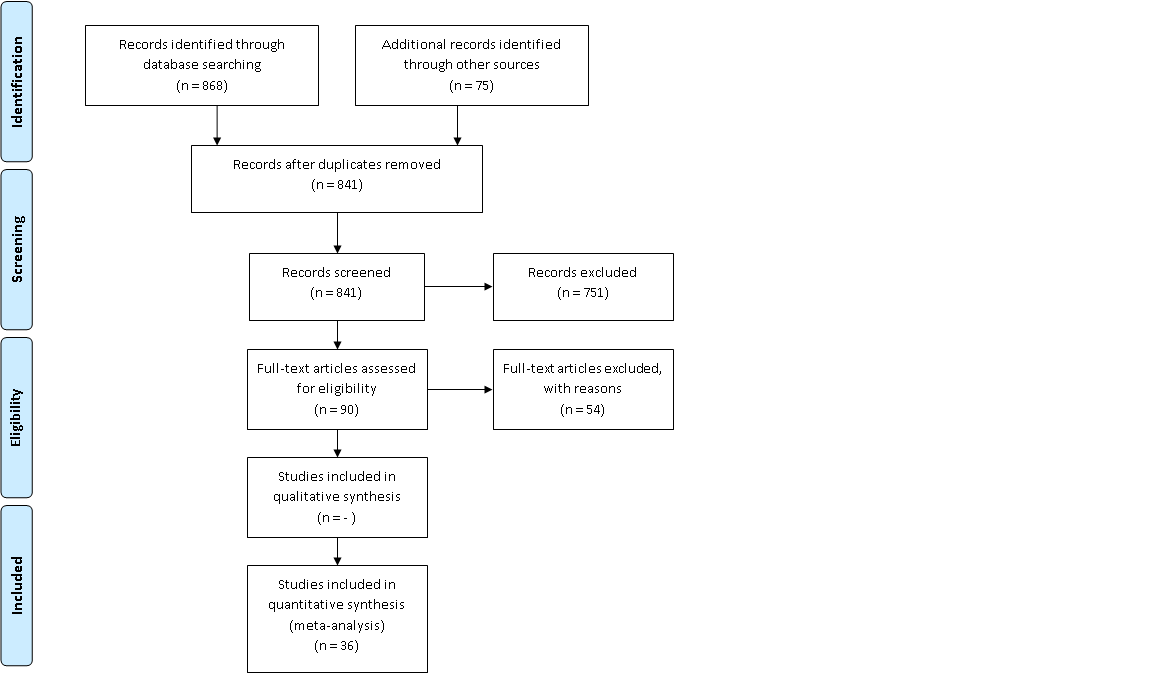

Supplement: Figure S1 — Prisma 2009 Flow Diagram. (TIF) [file pone.0104909.s001.tif]
