# Supplementary material for: A Systematic Review of Financial Debt in Adolescents and Young Adults: Prevalence, Correlates and Associations with Crime
Source: PLoS One. 2014 Aug 19;9(8):e104909. doi: 10.1371/journal.pone.0104909 (PMC4138212; doi:10.1371/journal.pone.0104909)
Supplement: Table S1 — Proportions of debt. (PDF) [file pone.0104909.s002.pdf]

Table S1

Proportions of debt

Study ID	Author (- study)	Year	*N*	Age group	Sample Type	Continent	% Females	% Ethnic minority	Year data collection	# Items	Type of debt	*ESp*
1	Blom, Weijters, and Van der Laan	2011	2,116	1	1	2	38.8	22.2	2010	4	3	.074
3	Davies and Lea	1995	140	2	2	2	50.0	9999	1992	4	1	.430
42	Dwyer, McCloud, and Hodson - NLSY79	2011	3,079	2	1	1	48.6	29.2	2004	1	2	.332
42	Dwyer, McCloud, and Hodson - NLSY79	2011	3,079	2	1	1	48.6	29.2	2004	1	12	.191
42	Henegar et al. - NLSY79	2013	2,618	2	1	1	51.0	9999	2008	1	2	.270
36	Hogan, Bryant, and Overymyer-Day	2013	338	2	2	1	58.0	14.0	2003	1	3	.230
42	Houle - NLSY97	2013	4,789	2	1	1	53.0	25.0	2007	1	12	.390
6	Jessop, Herberts, and Solomon	2005	187	2	2	2	69.5	9999	2003	1	1	.561
7	Johnes	1994	1,210	2	2	2	9999	9999	1992	1	12	.386
8	Joireman, Kees, and Sprott	2010	209	2	2	1	55.0	9999	2008	1	2	.354
9	Jones	2005	216	2	2	1	92.1	12.5	2002	2	1	.509
10	Lyons	2004	835	2	2	1	55.0	30.1	2001	5	3	.370
13	Nelissen, Van de Ven, and Stapel	2011	934	1	2	2	54.5	9999	2009	5	1	.246

14	Noorda	2008	73	3	3	2	26.0	92.3	2008	9999	1	.810
16	Norvilitis and MacLean	2010	173	2	2	1	77.5	26.6	2008	1	2	.665
18	Oosterbeek and Van den Broek	2009	5,621	2	2	2	71.2	9999	2007	1	1	.351
20	Robb and Sharpe	2009	3,884	2	2	1	65.8	13.7	2007	2	2	.250
21	Ross, Cleland, and MacLeod	2006	334	2	2	2	59.0	9999	2004	1	2	.224
21	Ross, Cleland, and MacLeod	2006	334	2	2	2	59.0	9999	2004	1	12	.790
21	Ross, Cleland, and MacLeod	2006	334	2	2	2	59.0	9999	2004	1	13	.156
21	Ross, Cleland, and MacLeod	2006	334	2	2	2	59.0	9999	2004	1	14	.536
22	Schwartz and Finnie	2002	80	2	2	1	53.0	9999	1990	1	3	.227
22	Schwartz and Finnie	2002	80	2	2	1	53.0	9999	1990	1	12	.459
23	Siennick - Add Health	2009	14,322	2	1	1	49.2	34.6	2003	1	2	.387
23	Siennick - Add Health	2009	14,322	2	1	1	49.2	34.6	2003	7	3	.320
23	Siennick - Add Health	2009	14,322	2	1	1	49.2	34.6	2003	1	12	.290
42	Siennick - NLSY79	2009	6,581	2	1	1	48.8	36.7	2002	1	1	.530
42	Siennick - NLSY79	2009	6,581	2	1	1	48.8	36.7	2002	1	11	.290
42	Siennick - NLSY79	2009	6,581	2	1	1	48.8	36.7	2002	1	12	.155
42	Siennick - NLSY79	2009	6,581	2	1	1	48.8	36.7	2002	1	15	.241
42	Siennick - NLSY79	2009	6,581	2	1	1	48.8	36.7	2002	1	16	.052
25	Van Dam	2005	57	3	3	2	0.0	9999	2001	1	1	.350

26	Van Heijst and Verhagen	2009	826	3	2	2	87.0	30.0	2007	1	1	.380
27	Wang and Xiao	2009	272	2	2	1	45.0	31.0	2007	1	2	.440
28	Zara and Farrington	2010	403	1	3	2	0.0	13.0	1970	9999	1	.241
29	Zhang and Kemp	2009	328	2	2	3	53.4	9999	2007	1	1	.896

Note. NLSY79 = National Longitudinal Survey of Youth 1997; Add Health = National Longitudinal Study of Adolescent Health; # Items = Number of items; *N* = number of participants; *ESp* = effect size proportion *p*. 9999 = missing value; Age group: 1 = adolescents, 2 = young adults, 3 = mixed; Sample type: 1 = general sample, 2 = students, 3 = high risk youths; Continent: 1 = North America, 2 = Europe, 3 = Australia; Type of debt: 1 = general debt, 2 = credit card debt, 3 = financial problems, 11 = consumer debt, 12 = student loan, 13 = bank loan, 14 = overdraft, 15 = auto loan, 16 = personal loan.
