# Supplementary material for: A Systematic Review of Financial Debt in Adolescents and Young Adults: Prevalence, Correlates and Associations with Crime
Source: PLoS One. 2014 Aug 19;9(8):e104909. doi: 10.1371/journal.pone.0104909 (PMC4138212; doi:10.1371/journal.pone.0104909)
Supplement: Table S2 — Correlates of debt. (PDF) [file pone.0104909.s003.pdf]

Table S2

Correlates of debt

Study ID	Author	Year	*N*	Type of debt	Domain	Correlate	*ESr*
31	Archuleta, Dale, and Spann	2013	180	1	1	Income	.070
31	Archuleta, Dale, and Spann	2013	180	1	1	Marital status	.120
31	Archuleta, Dale, and Spann	2013	180	1	1	Sex (female)	.150
31	Archuleta, Dale, and Spann	2013	180	1	1	Ethnic minority	-.070
31	Archuleta, Dale, and Spann	2013	180	1	5	Financial anxiety	.270
31	Archuleta, Dale, and Spann	2013	180	1	5	Financial well-being (vs. stress)	-.270
31	Archuleta, Dale, and Spann	2013	180	1	5	Financial knowledge	.180
42	Caputo	2012	5,304	1	1	Region (US south)	.060
42	Caputo	2012	5,304	1	1	Urbanization (urban)	.027
42	Caputo	2012	5,304	1	1	Ethnic minority	.168
42	Caputo	2012	5,304	1	1	SES	-.149
42	Caputo	2012	5,304	1	1	Sex (female)	.085
42	Caputo	2012	5,304	1	1	Age	-.066
42	Caputo	2012	5,304	1	2	Locus of control (externalizing)	.241
42	Caputo	2012	5,304	1	2	Self-esteem	-.377

42	Caputo	2012	5,304	1	3	Lived with parents	-.107
42	Caputo	2012	5,304	1	3	Change in parental income	.202
32	Crocker and Luhtanen	2003	631	3	2	Self-esteem	-.080
32	Crocker and Luhtanen	2003	631	3	2	Narcissism	.060
32	Crocker and Luhtanen	2003	631	3	2	Extraversion	.020
32	Crocker and Luhtanen	2003	631	3	2	Agreeability	-.040
32	Crocker and Luhtanen	2003	631	3	2	Conscientiousness	.020
32	Crocker and Luhtanen	2003	631	3	2	Neuroticism	.100
32	Crocker and Luhtanen	2003	631	3	2	Openness	.050
32	Crocker and Luhtanen	2003	631	3	2	School performance	-.190
32	Crocker and Luhtanen	2003	631	3	2	School performance	-.200
4	Grable and Joo	2006	110	2	1	Ethnic minority	.231
34	Ha	2013	257	2	1	Income	.363
34	Ha	2013	257	2	1	Marital status	.000
34	Ha	2013	257	2	1	Study year	.000
34	Ha	2013	257	2	1	Age	.000
5	Haultain, Kemp, and Chernyshenko	2010	125	1	2	Attitude towards deb (pro-debt)	.140
5	Haultain, Kemp, and Chernyshenko	2010	125	1	2	Attitude towards debt (pro-debt)	-.010
6	Jessop, Herberts, and Solomon	2005	187	1	1	Age	-.040

6	Jessop, Herberts, and Solomon	2005	187	1	1	Hours worked	.090
6	Jessop, Herberts, and Solomon	2005	187	1	2	Physical health	-.280
6	Jessop, Herberts, and Solomon	2005	187	1	2	Physical health	-.340
6	Jessop, Herberts, and Solomon	2005	187	1	2	locus of control (external)	-.060
6	Jessop, Herberts, and Solomon	2005	187	1	2	Physical health	-.160
6	Jessop, Herberts, and Solomon	2005	187	1	2	Physical health	-.070
6	Jessop, Herberts, and Solomon	2005	187	1	2	Mental health	-.130
6	Jessop, Herberts, and Solomon	2005	187	1	2	Social functioning	-.280
6	Jessop, Herberts, and Solomon	2005	187	1	2	Mental health	-.160
6	Jessop, Herberts, and Solomon	2005	187	1	2	Physical health	-.170
6	Jessop, Herberts, and Solomon	2005	187	1	2	Physical health	.170
6	Jessop, Herberts, and Solomon	2005	187	1	2	Physical health	-.260
6	Jessop, Herberts, and Solomon	2005	187	1	2	Physical health	-.060
6	Jessop, Herberts, and Solomon	2005	187	1	5	Financial wellbeing (vs. stress)	-.380
8	Joireman, Kees, and Sprott	2010	209	2	2	Take into account consequences of behavior	-.170
8	Joireman, Kees, and Sprott	2010	209	2	5	Compulsive buying	.510
12	Morra, Regehr, and Ginsburg	2008	549	1	1	Study year	.131
12	Morra, Regehr, and Ginsburg	2008	549	1	5	Financial wellbeing (vs. stress)	-.300
13	Nelissen, Van de Ven, and Stapel	2011	918	1	1	Income	.280

13	Nelissen, Van de Ven, and Stapel	2011	918	1	1	Age	.270
13	Nelissen, Van de Ven, and Stapel	2011	918	1	1	Sex (female)	.190
13	Nelissen, Van de Ven, and Stapel	2011	918	1	2	Self-esteem	-.040
13	Nelissen, Van de Ven, and Stapel	2011	918	1	4	Status concern	.070
13	Nelissen, Van de Ven, and Stapel	2011	918	1	4	Status concern	.060
13	Nelissen, Van de Ven, and Stapel	2011	918	1	4	Status concern	.110
13	Nelissen, Van de Ven, and Stapel	2011	918	1	4	Social comparison tendency	-.010
13	Nelissen, Van de Ven, and Stapel	2011	918	1	5	Attitude towards debt (pro-debt)	.120
13	Nelissen, Van de Ven, and Stapel	2011	918	1	5	Attitude towards debt (pro-debt)	.160
16	Norvilitis and MacLean	2010	173	2	2	Delay of gratification	-.100
16	Norvilitis and MacLean	2010	173	2	3	Parental financial support	-.080
16	Norvilitis and MacLean	2010	173	2	3	Parental financial support	-.250
16	Norvilitis and MacLean	2010	173	2	3	Parent worries	.040
16	Norvilitis and MacLean	2010	173	2	3	Parent talking about finances	-.020
16	Norvilitis and MacLean	2010	173	2	3	Parent bailout	-.290
16	Norvilitis and MacLean	2010	173	2	5	Financial knowledge	.200
16	Norvilitis and MacLean	2010	173	2	5	Financial wellbeing (vs. stress)	-.550
15	Norvilitis, Merwin, Osberg, Roehling, Young and Kamas	2011	398	2	1	Sex (female)	.049
15	Norvilitis, Merwin, Osberg, Roehling, Young and Kamas	2011	399	2	1	State school (vs. private school)	.155

18	Oosterbeek and Van den Broek	2009	5,621	1	1	Sex (female)	-.088
18	Oosterbeek and Van den Broek	2009	4,840	1	1	Age	.294
18	Oosterbeek and Van den Broek	2009	5,621	1	1	Holds part-time job	-.024
18	Oosterbeek and Van den Broek	2009	5,621	1	1	Hours worked	.023
18	Oosterbeek and Van den Broek	2009	5,621	1	1	Study hours	.030
18	Oosterbeek and Van den Broek	2009	5,621	1	1	Earnings after graduation	.000
18	Oosterbeek and Van den Broek	2009	5,621	1	1	Earnings after graduation	.009
18	Oosterbeek and Van den Broek	2009	5,621	1	1	Risk attitude	.114
18	Oosterbeek and Van den Broek	2009	2,623	1	2	School performance	-.007
18	Oosterbeek and Van den Broek	2009	4,784	1	2	School performance	-.005
18	Oosterbeek and Van den Broek	2009	5,621	1	2	School performance	-.131
18	Oosterbeek and Van den Broek	2009	5,621	1	2	School performance	-.064
18	Oosterbeek and Van den Broek	2009	5,621	1	2	probability to find suitable job	-.023
18	Oosterbeek and Van den Broek	2009	4,840	1	3	Parental income	-.076
18	Oosterbeek and Van den Broek	2009	4,764	1	3	Parents attitude to debt	.391
18	Oosterbeek and Van den Broek	2009	5,621	1	5	Discount rate (valuation of current vs. future consumption)	-.170
18	Oosterbeek and Van den Broek	2009	5,621	1	5	Attitude to debt (pro-debt)	.386
21	Ross, Cleland and MacLeod	2006	332	1	1	Sex (female)	.000

21	Ross, Cleland and MacLeod	2006	334	1	1	Study year	.438
21	Ross, Cleland and MacLeod	2006	334	1	1	SES	-.173
21	Ross, Cleland and MacLeod	2006	334	1	1	Hours worked	.000
21	Ross, Cleland and MacLeod	2006	334	1	2	School performance	.000
21	Ross, Cleland and MacLeod	2006	334	1	2	Mental health	-.096
21	Ross, Cleland and MacLeod	2006	334	1	5	Financial wellbeing (vs. stress)	-.127
24	Shim, Xiao, Barber, and Lyon	2009	781	1	1	Financial education	.030
24	Shim, Xiao, Barber, and Lyon	2009	781	1	1	Sex (female)	.040
24	Shim, Xiao, Barber, and Lyon	2009	781	1	1	Study year	.290
24	Shim, Xiao, Barber, and Lyon	2009	781	1	1	Income	.270
24	Shim, Xiao, Barber, and Lyon	2009	781	1	2	Self-esteem	.110
24	Shim, Xiao, Barber, and Lyon	2009	781	1	3	Parents talking about finances	-.110
24	Shim, Xiao, Barber, and Lyon	2009	781	1	3	Parental income	-.140
24	Shim, Xiao, Barber, and Lyon	2009	781	1	3	Willingness to meet parental expectations (towards positive financial behaviors)	.010
24	Shim, Xiao, Barber, and Lyon	2009	781	1	5	Perceived control toward financial management	.370
24	Shim, Xiao, Barber, and Lyon	2009	781	1	5	Financial knowledge	.050
24	Shim, Xiao, Barber, and Lyon	2009	781	1	5	Attitudes towards financial management	-.010
24	Shim, Xiao, Barber, and Lyon	2009	781	1	5	Financial management intention	-.180

24	Shim, Xiao, Barber, and Lyon	2009	781	1	5	Financial wellbeing (vs. stress)	.320
27	Wang and Xiao	2009	272	2	2	Seek social support	-.000
27	Wang and Xiao	2009	272	2	3	Parental income	-.000
27	Wang and Xiao	2009	272	2	5	Compulsive buying	.100
27	Wang and Xiao	2009	272	2	5	Compulsive buying	.100
27	Wang and Xiao	2009	272	2	5	Budget constraint	.100
29	Zhang and Kemp	2009	328	1	1	Sex (female)	.100
29	Zhang and Kemp	2009	328	1	1	Study year	.490
29	Zhang and Kemp	2009	328	1	2	Motivation towards study (intrinsic)	.150
29	Zhang and Kemp	2009	328	1	2	motivation towards study (intrinsic)	.160
29	Zhang and Kemp	2009	328	1	2	Study performance	-.060
29	Zhang and Kemp	2009	328	1	2	Study performance	.050
29	Zhang and Kemp	2009	328	1	2	Happiness	-.010
29	Zhang and Kemp	2009	328	1	5	Attitude towards debt (pro-debt)	.210
29	Zhang and Kemp	2009	328	1	5	Rated extent to which debt affects happiness	-.020

Note. Type of debt: 1 = general debt, 2 = credit card debt, 3 = financial problems; Domain: 1 = demographics; 2 = individual, 3 = family, 4 = peer, 5 = financial; *N* = number of participants; *ESr* = effect size correlation *r*.
